# A three-dimensional electrochemical oxidation system with α-Fe_2_O_3_/PAC as the particle electrode for ammonium nitrogen wastewater treatment

**DOI:** 10.1039/d0ra00032a

**Published:** 2020-02-28

**Authors:** Meng Yuan, Fangrong Yan, Yige Chen, Jujie Luo, Ziyan Li

**Affiliations:** State Key Laboratory of Soil Erosion and Dryland Farming on the Loess Plateau, Northwest A&F University Yangling 712100 PR China; College of Materials Science and Engineering, Taiyuan University of Technology Taiyuan 030024 PR China; College of Resources and Environment, Northwest A&F University Yangling 712100 PR China

## Abstract

A three-dimensional particle electrode loaded with α-Fe_2_O_3_ on powdered activated carbon (PAC) (α-Fe_2_O_3_/PAC) was synthesized by the microwave method for removing ammonium nitrogen from wastewater in a three-dimensional electrode system. The α-Fe_2_O_3_/PAC electrode was characterized by scanning electron microscopy (SEM) and X-ray diffraction (XRD). The effect of the added α-Fe_2_O_3_/PAC on the removal of ammonium nitrogen from simulated wastewater was studied by changing the cell voltage, particle dosage, and particle electrode synthesis conditions. Simulated experiments were also carried out on different pollutants under the best experimental conditions and the actual domestic sewage was tested. The results show that the optimal synthesis conditions of the particle electrode are as follows: the ratio of PAC to anhydrous FeCl_3_ is 1 : 2, and the microwave power is 1000 W for 60 s. After 20 min of electrolysis at 20 V, the ammonium nitrogen removal rate can reach 95.30%.

## Abstract 2

A microwave method was used to synthesis α-Fe_2_O_3_/PAC 3D particle electrode rapidly which can remove NH_4_^+^–N from wastewater.

## Graphical abstract

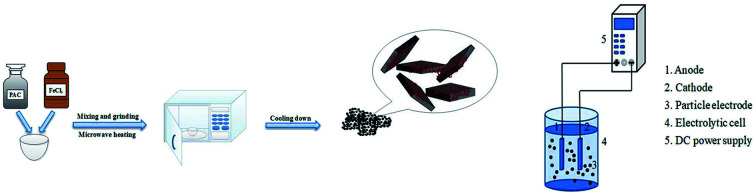

## 1. Introduction

Domestic sewage contains many pollutants, such as organic matter, phosphate, bacteria and ammonium nitrogen (NH_4_^+^–N).^1^ Particularly, as a significant nutrient, ammonium nitrogen can lead to eutrophication in polluted water when in excess. A high nutrient load gives rise to detrimental effects such as the rapid growth of harmful algae in contaminated water, reduced dissolved oxygen and declining water self-purification ability and can even compromise the structure and function of aquatic ecosystems.^2–5^ Hence, for reducing the negative impact of wastewater discharge, various traditional treatment methods have been studied to remove the ammonium nitrogen from polluted water, including biological treatments^6^ and diverse approaches of physical and chemical processes, such as adsorption,^7^ membrane filtration,^8^ ion exchange,^9^ air stripping,^10^ breakpoint chlorination^11^ and chemical precipitation.^12^

Electrochemical oxidation processes have been considered as environment friendly technologies in water treatment processes due to their strong oxidation properties, ease of control and mild reaction conditions.^13,14^ In effect, electrochemical technologies provide a second solution to numerous environmental issues in industrial processes because of electrons, which provide readily automatable, highly effective, economical, clean and multifunctional reagents.^15^ Three-dimensional (3D) electrodes have received wide attention due to the large specific surface area and high conversion rate compared with the conventional two-dimensional (2D) system. The reason for this phenomenon is that the tiny particles placed in the 3D electrode system create charged microelectrodes on account of the action of an electric field.^16,17^ Because of the large specific surface area of these particles, the 3D electrode system can adsorb contaminants, enhance the conductivity and even provide more active sites for the catalytic reaction, thereby increasing the removal efficiency.^18^ In the recent years, various conventional types of particle electrodes have been widely studied, including granular activated carbon, carbon aerogels, modified kaolin and metal particles.^17,19–21^

Iron oxide exists in many forms in nature, among which magnetite (Fe_3_O_4_), maghemite (γ-Fe_2_O_3_) and hematite (α-Fe_2_O_3_) are probably the most common and technically important.^22^ Iron oxides have been reported to have important applications in the removal of ammonium nitrogen. Zare *et al.* used Fe_3_O_4_ nanoparticles as adsorbents to remove ammonium ions from simulated wastewater. After 40 min of adsorption, the ammonium nitrogen removal rate was 94.3%.^23^ Wu *et al.* filled metal oxide (CuO, MnO_2_ and Fe_2_O_3_)-supported GAC as a particle electrode and studied the ability of the 3DER–3DBER system to eliminate nitrogen contaminants.^24^ Liu *et al.* achieved an ammonium removal rate of 89.97% after adsorption for 2 hours using magnetic zeolite NaA with 3.4% Fe_3_O_4_ loading.^25^ In addition, AC has been applied as a carrier material because of its excellent adsorption ability, porous structure and mechanical strength.^26^ Therefore, the purpose of this study was to synthesize a modified powdered activated carbon particle electrode loaded with α-Fe_2_O_3_ (α-Fe_2_O_3_/PAC) by the microwave method in one step, characterize the α-Fe_2_O_3_/PAC electrode, and analyze how the synthesized α-Fe_2_O_3_/PAC as the particle electrode participates in the degradation of NH_4_^+^–N in simulated wastewater.

## 2. Materials and methods

### 2.1 Materials

Powdered activated carbon (PAC) was purchased from Gongyi Starfish Water Supply Material Co. Ltd. Anhydrous FeCl_3_ was supplied by Shanghai Aladdin Biochemical Technology Co. Ltd. The other chemicals were obtained from Tianjin Kaitong Chemical Reagent Co. Ltd and were of analytical grade. Deionized water was used in all experiments.

NH_4_^+^–N simulated wastewater with a concentration of 140 mg L^−1^ was prepared by dissolving NH_4_Cl in deionized water. Total nitrogen (TN) simulated wastewater with a concentration of 200 mg L^−1^ was prepared by mixing NH_4_Cl solution and KNO_3_ solution, wherein the NH_4_^+^–N concentration was 140 mg L^−1^ and NO_3_^+^–N was 60 mg L^−1^. The total phosphorus (TP) simulation solution of 5 mg L^−1^ was prepared from KH_2_PO_4_. The actual wastewater used in this study was the domestic sewage of a factory in China. Some of the characteristics of the domestic wastewater used in the experiment are shown in Table 1.

**Table 1:** Characteristics of the domestic wastewater used in the experiment

NH_4_^+^–N	TN	TP	COD
64.50 mg L^−1^	72.68 mg L^−1^	4.47 mg L^−1^	49.66 mg L^−1^

### 2.2 Preparation of particle electrodes

**Fig. 1:**
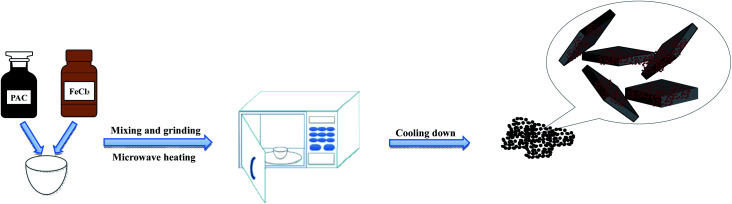
Synthesis flow chart of particle electrodes.

PAC and anhydrous FeCl_3_ were mixed in different mass ratios and ground uniformly using an agate mortar. The mixture transferred from the mortar to a crucible was subjected to a microwave reaction in a household microwave oven (Microwave Oven, Panasonic NN-GF352M, 2450 MHz, 1000 W) with different heating times and microwave powers. After cooling for 1 hour, the obtained particle electrodes were stored in glass bottles. The synthesis flow chart is shown in Fig. 1. Table 2 shows the different experimental conditions for preparing the α-Fe_2_O_3_/PAC particle electrodes.

**Table 2:** Different experimental conditions for preparing α-Fe_2_O_3_/PAC particle electrodes

Sample	PAC : FeCl_3_	Microwave power (W)	Heating time (s)
a	1 : 1	1000	60
b	1 : 2	1000	60
c	1 : 3	1000	60
d	2 : 1	1000	60
e	1 : 2	1000	30
f	1 : 2	1000	90
g	1 : 2	800	60

### 2.3 Experimental procedure

**Fig. 2:**
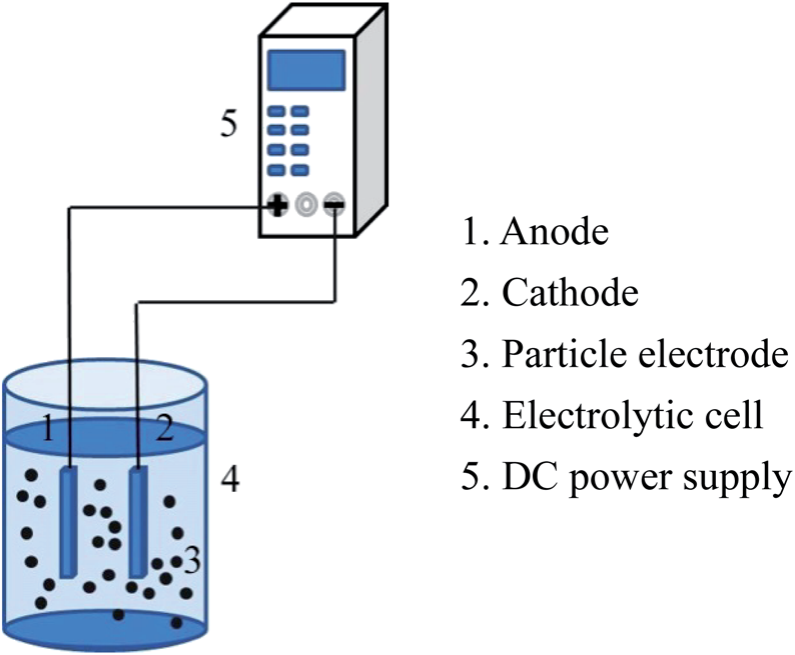
Experimental device.

The experimental device of the 3D electrode system is shown in Fig. 2. 3D electrochemical oxidation experiments were performed in a heat-resistant glass tank with an effective volume of 85 mL. Ti/RuO_2_–IrO_2_ electrode (25 mm × 20 mm × 1 mm) (Baoji Jinbu Titanium Nickel Equipment Co. Ltd) and stainless steel plate (25 mm × 20 mm × 1 mm) were employed as the anode and cathode. The two plates were parallel to each other with a spacing of 2 cm. The power source used in the experiment was regulated DC power supply (APS3005DM, ATTEN Instrument, China). A certain mass of the particle electrode was added to the system for electrolysis experiment under the corresponding voltage. All the experiments were performed at room temperature, and 2 mL water samples were taken from the system every 10 min for testing the corresponding indicators.

### 2.4 Analysis

NH_4_^+^–N, TN, TP and COD were measured according to the Chinese National Standard Methods (HJ 535-2009, HJ 636-2012, GB 11893-89 and HJ 828-2017, respectively). NH_4_^+^–N concentration was tested by a UV-visible spectrophotometer (UV-9000s, Shanghai Yuanxi Instrument Co. Ltd, China). TN, TP and COD concentrations were measured by a rapid water quality tester (LH-3BA, Lanzhou Lianhua Environmental Protection Technology Co. Ltd, China). The synthesized material was analyzed by a field emission scanning electron microscope (SEM, JSM-7200F, China) and X-ray diffraction (XRD, DX2700B, China).

The removal efficiency of the pollutants (NH_4_^+^–N, TN, TP and COD) at time *t* can be calculated using the following eqn (1):1
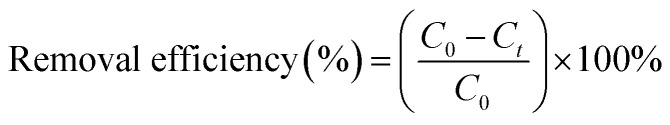
Here, *C*_0_ (mg L^−1^) is the initial concentration of the pollutant, and *C*_*t*_ (mg L^−1^) is the concentration of the pollutant at time *t*.

## 3. Results and discussion

### 3.1 Characterization of α-Fe_2_O_3_/PAC particle electrode

**Fig. 3:**
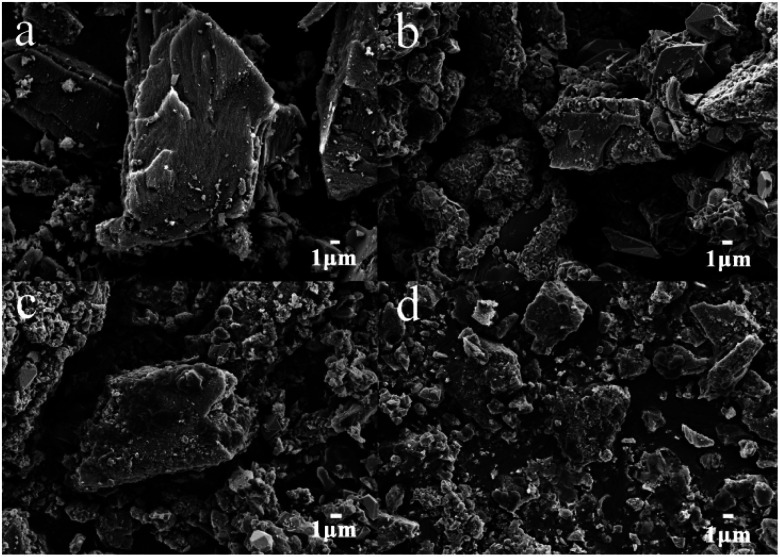
SEM images of (a) PAC, (b) α-Fe_2_O_3_/PAC (sample b), (c) α-Fe_2_O_3_/PAC (sample a) and (d) α-Fe_2_O_3_/PAC (sample f).

The characteristics of the synthesized α-Fe_2_O_3_/PAC particle electrode were investigated by SEM and XRD. Fig. 3 presents the SEM images of the particle electrode magnified 3000 times. The SEM image in Fig. 3(a) shows the morphology of PAC. The surface of the PAC particles exhibits a relatively smooth state. As shown in Fig. 3(b–d), the materials grown on the surface of the PAC particles are approximately spherical and irregularly polyhedral due to the loading of α-Fe_2_O_3_. Different synthesis conditions may result in different particle morphologies of the samples. The material shown in Fig. 3(b) displays good regularity and crystallinity in the crystal structure of α-Fe_2_O_3_ (sample b). The samples depicted in Fig. 3(c and d) (sample a and sample f) show poor crystallinity and insufficient loading on PAC as a result of unsuitable material synthesis conditions.

**Fig. 4:**
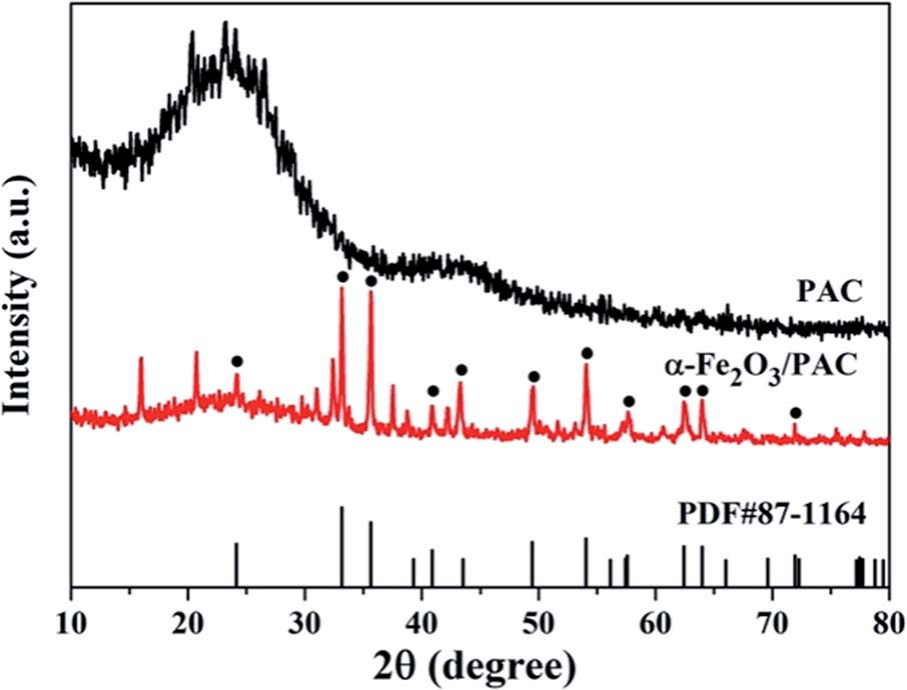
XRD patterns of PAC and α-Fe_2_O_3_/PAC (sample b).

As shown in Fig. 4, the synthesized samples of the α-Fe_2_O_3_/PAC materials (sample b) under optimal synthesis conditions and PAC have been characterized by XRD. The XRD pattern of PAC in Fig. 4 shows that PAC exhibits two dispersion peaks. One of them is a significantly broad peak at approximately 2*θ* = 23°, which also appears in the diffraction pattern of the synthesized sample (b). The diffraction peaks of α-Fe_2_O_3_/PAC were consistent with the standard hematite (α-Fe_2_O_3_) pattern (PDF#87-1164). The main peaks were located at 2*θ* = 24.16°, 33.16°, 35.62°, 40.84°, 43.3°, 49.54°, 54.1°, 57.64°, 62.56°, 64°, and 71.92°, corresponding to the (012), (104), (110), (113), (202), (024), (116), (018), (214), (300) and (1010) planes.

### 3.2 Effect of cell voltage

The chloride ions in water are oxidized to Cl_2_ molecules at the anode, which then dissolve in water to form HClO. HClO reacts with NH_4_^+^ to form N_2_.^11^ The main reactions are shown in the following equations:^27^

2 Cl_2_ + H_2_O → HClO + H^+^ + Cl^−^

3 2NH_4_^+^ + 3HClO → N_2_ + 3H_2_O + 5H^+^ + 3Cl^−^

**Fig. 5:**
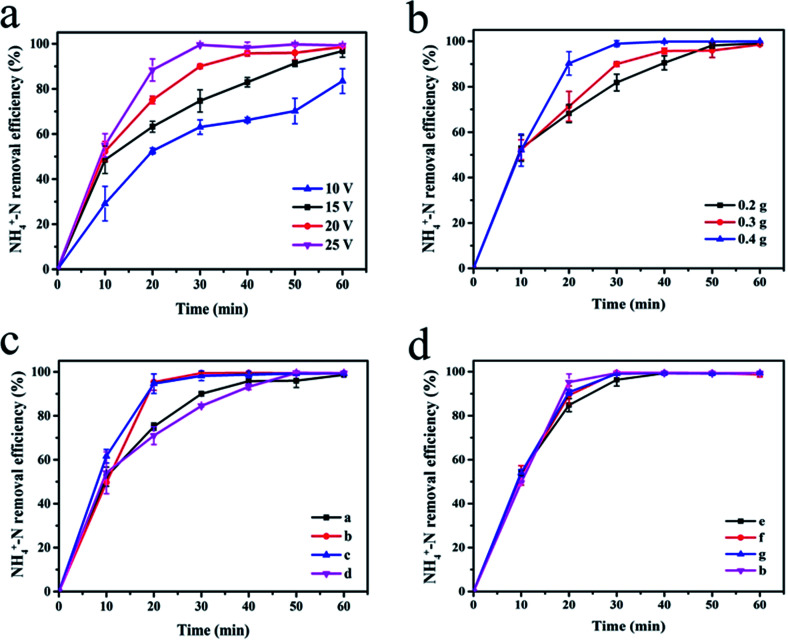
Degradation of NH_4_^+^–N under different experimental conditions ((a) at different cell voltage; (b) at different dosage of α-Fe_2_O_3_/PAC; (c and d) at different synthesis conditions of α-Fe_2_O_3_/PAC).

Fig. 5(a) shows the effect of cell voltage on the degradation efficiency of NH_4_^+^–N. Experiments were carried out by adding 0.3 g of the synthesized sample (a) as the particle electrode of the 3D electrode system. As the voltage increased, the NH_4_^+^–N removal rate also increased. After electrolysis at 10 V and 15 V for 40 min, the NH_4_^+^–N removal rates were 66.14% and 82.95%, respectively. At 20 V and 25 V, the degradation rate curves tend to be stable after 40 min of electrolysis. At this time, the electrolysis of NH_4_^+^–N tended to saturate, and the degradation rate (>95%) no longer increased considerably. At the appropriate voltage, particles can be polarized to form a myriad of microelectrodes, which can enhance the oxidation and increase the rate of the above-mentioned reactions.^18^ As the cell voltage increases, the number of polarized particles rise, which leads to an enhancement in the rate of oxidation and improvement in the rate of degradation. In addition, after 20 min of electrolysis using 20 V and 25 V as the battery voltage, the NH_4_^+^–N removal efficiency exceeded 95%. However, a very high voltage will cause an increase in the system temperature, resulting in heat loss and energy waste.

### 3.3 Effect of the amount of α-Fe_2_O_3_/PAC particle electrode

The change in the NH_4_^+^–N degradation rate at different dosage of the α-Fe_2_O_3_/PAC particles (voltage = 20 V) is shown in Fig. 5(b). The increase in the amount of α-Fe_2_O_3_/PAC added led to an increase in the rate of degradation. This phenomenon became apparent after 10 min of electrolysis and after 30 min, the degradation curve after adding 0.4 g α-Fe_2_O_3_/PAC particles first started to stabilize, at which time the electrolysis was saturated. However, at the same time, the removal efficiencies after adding 0.2 g and 0.3 g α-Fe_2_O_3_/PAC particles were 81.89% and 90.00%, respectively. When a certain voltage was applied to both sides of the electrolytic cell, each α-Fe_2_O_3_/PAC particle electrode in the cell was polarized and charged and served as the anode and cathode of the micro-electrolytic cell. Moreover, the adsorption of activated carbon makes oxidation more likely to occur. As the number of added particle electrodes increased, more charged microelectrodes were formed, resulting in an increase in electrolysis efficiency.^28–30^ Furthermore, after 40 min of electrolysis, the NH_4_^+^–N removal efficiency was as high as 95% after adding 0.3 g and 0.4 g α-Fe_2_O_3_/PAC particles. At this point, the increase in dosage will not lead to a significant increase in the efficiency of degradation.

### 3.4 Effect of preparation conditions of particle electrode

The effects of different synthesis conditions of particle electrodes on NH_4_^+^–N removal in a 3D electrode system are shown in Fig. 5(c and d).

A large proportion of FeCl_3_ in the synthesis process led to better degradation (Fig. 5(c)). After 20 min of electrolysis, the electrolysis efficiency of the experiment using samples (b) and (c) reached 95%, showing a significantly higher level than that for sample (a) (75.08%) and sample (d) (70.96%). This phenomenon can be explained by the fact that as the content of Fe_2_O_3_ in the synthesized particle electrodes increases, α-Fe_2_O_3_/PAC can form more micro-electrolytic cells, resulting in a larger specific surface area and more efficient mass transfer distance. The smaller distance between the anode and cathode in each micro-electrolysis cell is more beneficial for the transfer of reagents between the electrode surfaces.^28^ Inevitably, more Fe_2_O_3_ will lead to the dissolution of more iron ions to some extent. The possibility of the formation of iron hydroxide rises, which will improve the removal efficiency of NH_4_^+^–N in cooperation with three-dimensional electrolysis. The specific synthesis conditions are shown for the samples (a–d) in Table 2. In addition, the effect of the particle electrodes reacted at different microwave powers and times on the degradation rate of NH_4_^+^–N is not much different, and sample (b) shows the best effect (Fig. 5(d)). Short microwave heating times and inadequate microwave power may result in incomplete material synthesis. The SEM image shown in Fig. 3(c and d) indicates insufficient Fe_2_O_3_ supported on the activated carbon. Table 3 shows the degradation of NH_4_^+^–N after electrolysis for 20 min at 20 V under different particle electrode preparation conditions.

**Table 3:** Degradation of NH_4_^+^–N after electrolysis for 20 min under different particle electrode preparation conditions

Sample	a	b	c	d	e	f	g
NH_4_^+^–N degradation rate (%)	75.08	95.30	94.61	70.96	84.80	89.33	90.67

### 3.5 Experiment under optimal electrolysis conditions

**Fig. 6:**
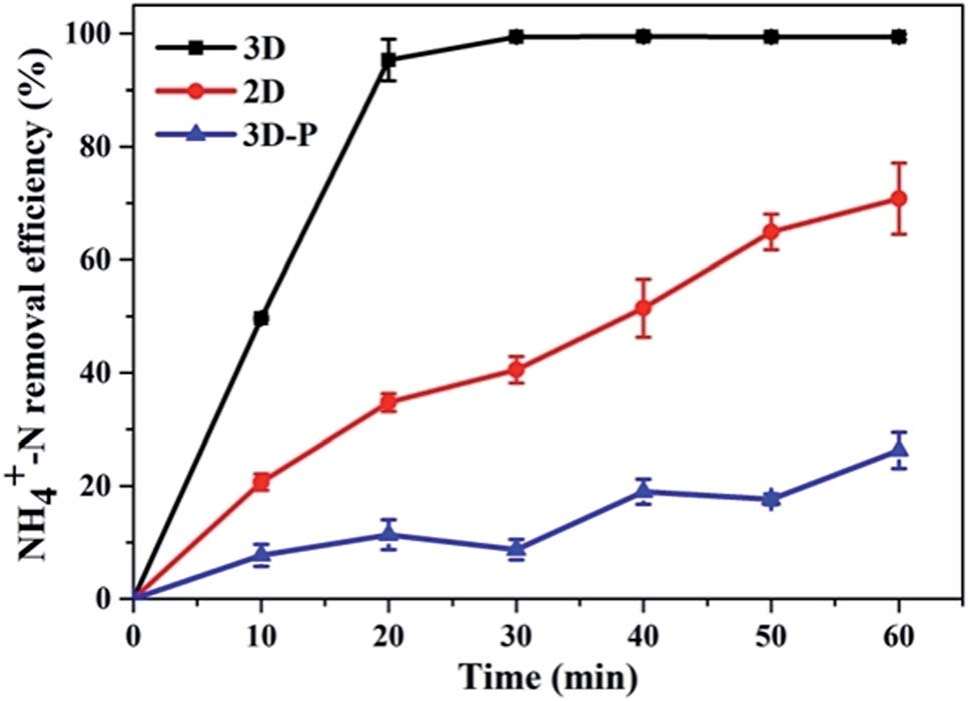
Degradation of NH_4_^+^–N (2D, 3D and 3D-P) under optimal experimental conditions (conditions: particle electrode = 0.3 g sample (b); voltage = 20 V).

**Fig. 7:**
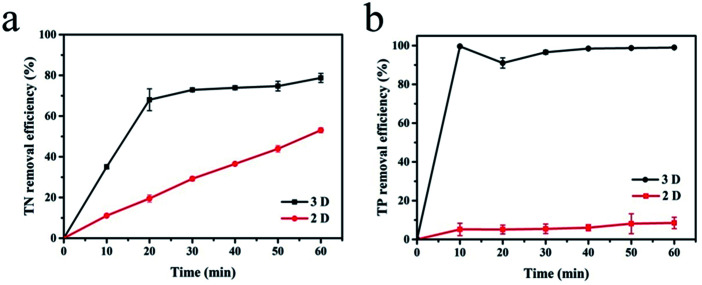
Degradation of (a) TN and (b) TP (2D and 3D) under optimal experimental conditions (conditions: particle electrode = 0.3 g sample (b); voltage = 20 V).

With sample (b) as the particle electrode and a voltage of 20 V, a 3D electrolysis experiment was carried out, and NH_4_^+^–N, TN and TP were studied under 2D and 3D system conditions using simulated wastewater. It can be clearly observed in Fig. 6 and 7 that the NH_4_^+^–N, TN and TP removal efficiency in the 2D system rises slowly with the increase in time and in the 3D system, it rapidly rises first and then maintains a smooth state. In particular, PAC was used as the particle electrode for the electrolytic testing of NH_4_^+^–N under the same 3D conditions (3D-P). The results showed that under the influence of activated carbon adsorption, low impedance and weak catalytic performance, the degradation curve increased tortuously, and the removal efficiency of NH_4_^+^–N after 60 min of electrolysis was only 26.27%. The degradation of NH_4_^+^–N (Fig. 6) and TN (Fig. 7(a)) increased rapidly in the first 20 min and then did not increase significantly. The degradation efficiency of NH_4_^+^–N was 95.30% in the 3D system but only 34.73% in the 2D system after treatment for 20 min. In the same case, the removal efficiencies of TN were 67.97% (3D) and 19.47% (2D). Besides, TP degraded in 10 min (99.59%) in the 3D system, and only 5.15% TP degraded in the 2D system (Fig. 7(b)).

The oxidation and removal of ammonium nitrogen by HClO during the electrolysis process play leading roles in this study. The main mechanism of ammonium nitrogen degradation is the secondary oxide produced by anodic electrolysis to remove NH_4_^+^–N.^31,32^ However, a small amount of iron hydroxide precipitated during the electrolysis process also plays a role in the removal of ammonium nitrogen. The reason behind this phenomenon is that when the ammonium nitrogen removal effect is remarkable, the amounts of the oxidizing substance (HClO) and H^+^ in the solution are large, resulting in a decrease in the pH of the solution.^33^ At the same time, a small amount of an electrolyzed water side reaction may occur during electrolysis. It has been shown in the literature that at pH < 5.6, the dominant functional group on the surface of iron oxide is Fe^2+^ or FeOH^+^, and iron oxide attracts anions at low pH. When the ambient pH is lower than the pH_PZC_ (6.7) of hematite, anions are expected to adsorb on the surface of the positively charged hematite by electrostatic attraction.^34–36^ The above-mentioned explanation can be regarded as one of the reasons why TP can be removed in large quantities.

### 3.6 Effect of actual domestic wastewater degradation

The experimental system can obtain a large NH_4_^+^–N removal rate in a short time (20 min) under optimal conditions, and its application for the actual domestic sewage is also effective. Based on the exploration of the above-mentioned experiments, an electrolysis experiment was carried out on the domestic sewage of a factory. The detailed degradation efficiency data under the three-dimensional system are shown in Table 4. Due to the complexity of the components in the actual sewage, the oxidizing substances in the water are more complicated than that in the simulated wastewater. Therefore, after 20 min of electrolytic treatment, the degradation effect of NH_4_^+^–N in the sewage can reach 98.97%. Meanwhile, TN, TP and COD in the wastewater were also tested. It was found that the removal rate of TN was 91.11% after 20 min, and TP achieved good results after 10 min with a removal rate of 99.75%. COD also had a removal rate of 52.88% after 40 min of electrolysis. Compared with other studies for the removal of NH_4_^+^–N, this study can achieve a higher ammonium nitrogen removal rate in a shorter period of time (Table 5).

**Table 4:** Three-dimensional electrode method for removing pollutants from domestic sewage under optimal experimental conditions

Parameter	NH_4_^+^–N (20 min)	TN (20 min)	TP (10 min)	COD (40 min)
Before treatment (mg L^−1^)	64.50	72.68	4.47	49.66
Degradation rate (%)	98.97	91.11	99.75	52.88

**Table 5:** Summary of some research data on ammonia removal

Method	Anode and cathode	Wastewater	Experiment conditions	Time	Results	References
3D electrochemical oxidation	Ti/RuO_2_–IrO_2_; stainless steel	NH_4_Cl solution	Voltage: 20 V; 0.3 g α-Fe_2_O_3_/PAC particle electrode	20 min/30 min	NH_4_^+^–N removal: 95.30%/99%	This article
3D electrochemical oxidation	Ti/RuO_2_–IrO_2_; stainless steel	Domestic sewage	Voltage: 20 V; 0.3 g α-Fe_2_O_3_/PAC particle electrode	20 min	NH_4_^+^–N removal: 98.97%	This article
Biological pretreatment and electrochemical oxidation	Ti/RuO_2_–IrO_2_; stainless steel	Coking plant wastewater	Current density: 15.46 mA cm^−2^	60 min	NH_4_^+^–N removal: 95.23–99.35%	14
Electrochemical process and ultraviolet irradiation	RuO_2_/Ti or IrO_2_/Ti mesh; Ti mesh	NH_4_Cl/NaCl simulated wastewater	Current density: 40 mA cm^−2^; Cl^−^ concentration 5300 mg L^−1^; UV irradiation	120 min	NH_4_^+^–N conversion: 98%	39
Chemical precipitation	—	NH_4_Cl solution	Stirred at 120 rpm	80 min	NH_4_^+^ removal: 76.63%	40
Adsorption	—	Ammonium chloride salt	Add Fe_3_O_4_ nanoparticles; ultrasonic bath; *T* = 298 K	40 min	NH_4_^+^ removal: 93.12%	23
Photocatalytic	—	NH_4_Cl solution	Catalyst: TiO_2_–CuO/HSC; UV irradiation	120 min	NH_4_^+^ removal: 99.7%	41

Since the particle electrode used in this study was synthesized with PAC as the carrier, the separation of PAC in practical applications is also a crucial topic that needs to be discussed. According to recent research, several separation processes have been widely used in practical plants, such as sedimentation, membrane filtration, ultrafiltration and pile cloth filtration. It has been reported that flocculation may have given PAC a better settling characteristic with the addition of iron.^37^ For this reason, α-Fe_2_O_3_/PAC can be separated by sedimentation in practical applications. However, the hydraulic retention time required for sedimentation needs to be greater than 120 min. Thus, in practical applications, combining precipitation with membrane filtration (backwash interval = 10–20 minutes) can be considered to improve the processing efficiency.^38^

## 4. Conclusion

It is beneficial to use α-Fe_2_O_3_/PAC as a 3D particle electrode for the electrochemical oxidation of ammonium nitrogen. The removal rate of ammonium nitrogen in the electrolysis for 20 min is as high as 95.30% from the simulated wastewater under the optimal conditions (an applied voltage of 20 V, an α-Fe_2_O_3_/PAC dosage of 0.3 g (PAC : FeCl_3_ = 1 : 2, microwave power = 1000 W, time = 60 s)). Under identical conditions, after the domestic sewage was tested, the removal efficiencies of NH_4_^+^–N and TN were found to be 98.97% and 91.11%, respectively, at the end of 20 min. Moreover, TP removal could reach 99.87% in 10 min. Consequently, the 3D electrochemical oxidation with the α-Fe_2_O_3_/PAC particle electrode is a prospective process for NH_4_^+^–N wastewater treatment.

## Conflicts of interest

There are no conflicts to declare.

## Supplementary Material

## References

[cit1] Cheng H., Zhu Q., Xing Z. (2019). J. Cleaner Prod..

[cit2] Cheng Y., Huang T., Shi X., Wen G., Sun Y. (2017). J. Environ. Sci..

[cit3] Huang J., Kankanamge N. R., Chow C., Welsh D. T., Li T., Teasdale P. R. (2018). J. Environ. Sci..

[cit4] Huang H., Huang L., Zhang Q., Jiang Y., Ding L. (2015). Chemosphere.

[cit5] Liang Z., Li S., Guo W., Fan C. (2011). Chin. J. Chem. Eng..

[cit6] Abu Hasan H., Sheikh Abdullah S. R., Kamarudin S. K., Tan Kofli N., Anuar N. (2014). Sep. Purif. Technol..

[cit7] Le Leuch L. M., Bandosz T. J. (2007). Carbon.

[cit8] Hasanoğlu A., Romero J., Pérez B., Plaza A. (2010). Chem. Eng. J..

[cit9] Lin S. H., Wu C. L. (1996). Water Res..

[cit10] Yuan M.-H., Chen Y.-H., Tsai J.-Y., Chang C.-Y. (2016). Process Saf. Environ. Prot..

[cit11] Pressley T. A., Bishop D. F., Roan S. G. (1972). Environ. Sci. Technol..

[cit12] Zhang T., Ding L., Ren H., Xiong X. (2009). Water Res..

[cit13] Zhu X., Ni J., Lai P. (2009). Water Res..

[cit14] He X., Chai Z., Li F., Zhang C., Li D., Li J., Hu J. (2013). J. Chem. Technol. Biotechnol..

[cit15] Martínez-Huitle C. A., Panizza M. (2018). Curr. Opin. Electrochem..

[cit16] Huang J., Ming Y., Du Y., Wang Y., Wang C. (2016). J. Anal. Methods Chem..

[cit17] Kong W., Wang B., Ma H., Gu L. (2006). J. Hazard. Mater..

[cit18] Zhang C., Jiang Y., Li Y., Hu Z., Zhou L., Zhou M. (2013). Chem. Eng. J..

[cit19] Wang L., Fu J., Qiao Q., Zhao Y. (2007). J. Hazard. Mater..

[cit20] Yan L., Ma H., Wang B., Wang Y., Chen Y. (2011). Desalination.

[cit21] Wu X., Yang X., Wu D., Fu R. (2008). Chem. Eng. J..

[cit22] Teja A. S., Koh P.-Y. (2009). Prog. Cryst. Growth Charact. Mater..

[cit23] Zare K., Sadegh H., Shahryari-ghoshekandi R., Asif M., Tyagi I., Agarwal S., Gupta V. K. (2016). J. Mol. Liq..

[cit24] Wu Z. Y., Liu Y., Wang S. Y., Peng P., Li X. Y., Xu J., Li W. H. (2019). Bioresour. Technol..

[cit25] Liu H., Peng S., Shu L., Chen T., Bao T., Frost R. L. (2013). J. Colloid Interface Sci..

[cit26] Zhang C., Zhou L., Yang J., Yu X., Jiang Y., Zhou M. (2014). Environ. Sci. Pollut. Res..

[cit27] Kalaruban M., Loganathan P., Kandasamy J., Naidu R., Vigneswaran S. (2017). Sep. Purif. Technol..

[cit28] Zheng T., Wang Q., Shi Z., Fang Y., Shi S., Wang J., Wu C. (2016). J. Environ. Sci..

[cit29] Zhu X., Ni J., Xing X., Li H., Jiang Y. (2011). Electrochim. Acta.

[cit30] Sun H., Chen T., Kong L., Cai Q., Xiong Y., Tian S. (2015). Ind. Eng. Chem. Res..

[cit31] Kim K.-W., Kim Y.-J., Kim I.-T., Park G.-I., Lee E.-H. (2005). Electrochim. Acta.

[cit32] Chiang L.-C., Chang J.-E., Wen T.-C. (1995). Water Res..

[cit33] Kim K. W., Kim Y. J., Kim I. T., Park G. I., Lee E. H. (2006). Water Res..

[cit34] Zhu Z., Zeng H., Zhu Y., Yang F., Zhu H., Qin H., Wei W. (2013). Sep. Purif. Technol..

[cit35] Fang H., Cui Z., He G., Huang L., Chen M. (2017). Sci. Total Environ..

[cit36] Sverjensky D. A. (1994). Geochim. Cosmochim. Acta.

[cit37] Luo Y., Guo W., Ngo H. H., Nghiem L. D., Hai F. I., Zhang J., Liang S., Wang X. C. (2014). Sci. Total Environ..

[cit38] Krahnstöver T., Wintgens T. (2018). J. Environ. Chem. Eng..

[cit39] Xiao S., Qu J., Zhao X., Liu H., Wan D. (2009). Water Res..

[cit40] Si Q., Zhu Q., Xing Z. (2018). Ecotoxicol. Environ. Saf..

[cit41] Peng X., Wang M., Hu F., Qiu F., Dai H., Cao Z. (2019). J. Alloys Compd..

